# Wnt/β-Catenin Signaling Modulates Human Airway Sensitization Induced by β_2_-Adrenoceptor Stimulation

**DOI:** 10.1371/journal.pone.0111350

**Published:** 2014-10-31

**Authors:** Christophe Faisy, Stanislas Grassin-Delyle, Sabine Blouquit-Laye, Marion Brollo, Emmanuel Naline, Alain Chapelier, Philippe Devillier

**Affiliations:** 1 Unité Propre de Recherche de l'Enseignement Supérieur, Equipe d'Accueil 220, Université Versailles Saint–Quentin, Hôpital Foch, Suresnes, France; 2 Medical Intensive Care Unit, Hôpital Européen Georges Pompidou, Assistance Publique-Hôpitaux de Paris, Paris, France; 3 Department of Thoracic Surgery, Hôpital Foch, Suresnes, France; University of Kentucky, United States of America

## Abstract

**Background:**

Regular use of β_2_-agonists may enhance non-specific airway responsiveness. The wingless/integrated (Wnt) signaling pathways are responsible for several cellular processes, including airway inflammation and remodeling while cAMP–PKA cascade can activate the Wnt signaling. We aimed to investigate whether the Wnt signaling pathways are involved in the bronchial hyperresponsiveness induced by prolonged exposure to β_2_-adrenoceptor agonists in human isolated airways.

**Methods:**

Bronchi were surgically removed from 44 thoracic surgery patients. After preparation, bronchial rings and primary cultures of bronchial epithelial cells were incubated with fenoterol (0.1 µM, 15 hours, 37°C), a β_2_-agonist with high intrinsic efficacy. The effects of inhibitors/blockers of Wnt signaling on the fenoterol-induced airway sensitization were examined and the impact of fenoterol exposure on the mRNA expression of genes interacting with Wnt signaling or cAMP–PKA cascade was assessed in complete bronchi and in cultured epithelial cells.

**Results:**

Compared to paired controls, fenoterol-sensitization was abolished by inhibition/blockage of the Wnt/β-catenin signaling, especially the cell-surface LRP5/6 co-receptors or Fzd receptors (1 µM SFRP1 or 1 µM DKK1) and the nuclear recruitment of TCF/LEF transcriptions factors (0.3 µM FH535). Wnt proteins secretion did not seem to be involved in the fenoterol-induced sensitization since the mRNA expression of Wnt remained low after fenoterol exposure and the inactivator of Wnt secretion (1 µM IWP2) had no effect on the fenoterol-sensitization. Fenoterol exposure did not change the mRNA expression of genes regulating Wnt signaling or cAMP–PKA cascade.

**Conclusions:**

Collectively, our pharmacological investigations indicate that fenoterol-sensitization is modulated by the inhibition/blockage of canonical Wnt/β-catenin pathway, suggesting a phenomenon of biased agonism in connection with the β_2_-adrenoceptor stimulation. Future experiments based on the results of the present study will be needed to determine the impact of prolonged fenoterol exposure on the extra- and intracellular Wnt signaling pathways at the protein expression level.

## Introduction

Wnt (wingless/integrated) is a large family of secreted glycoproteins with highly conserved cysteine residues involved in lung development and diseases [1]. The *WNT* gene family includes 19 members encoding Wnts, which can activate three distinct signaling pathways. The best characterized canonical Wnt/β-catenin pathway implicated the inhibition of glycogen synthase kinase GSK-3β, resulting a cytoplasmic accumulation of β-catenin and its nuclear translocation [2]–[4]. The two non-canonical Wnt pathways do not require β-catenin as a co-transcription factor [4], [5]. Therefore, the Wnt/Ca^2+^ signaling pathway is mediated by protein kinase C (PKC) and the Wnt/planar cell polarity (PCP) pathway activates the small G proteins Rho and the mitogen-activated proteins kinases (MAPK) cascade or alternatively triggers activation of the c-Jun-N-terminal kinase (JNK) leading to the transcription of target genes through the activator protein-1 (AP-1) stimulation [1], [2], [5]. Wnts are expressed in the distal mesenchyme and in airway epithelium and act via the seven membrane-spanning Fzd cell-surface receptors [1], [2], [6], [7]. The Fzd family includes 10 distinct members [1], [4], [7], most of which can activate β-catenin signaling when combined with the lipoprotein-related co-receptors LRP5/6 [2], [8]. The Wnt-induced phosphorylation of LRP is critical for Fzd-LRP association [2], [8]. Unlike the canonical pathways, the Wnt/Ca^2+^ and the Wnt/PCP signaling pathways are independent from LRP5/6 [2], [8]. The Wnt signaling pathways are responsible for several cellular processes, including cell movement and polarity, proliferation and differentiation of the airway epithelium, airway epithelial repair and cytoskeletal reorganization after airway stretching [1], [4], [7], [9]–[12]. Wnts also exert autocrine-signaling activity on airway epithelial cells [1], [6]. Depending on the cellular context, Wnts stimulate the canonical signaling pathway, thereby up-regulating inflammatory genes such as cyclo-oxygenase 2 (COX-2), interleukine-8 (IL-8), and matrix metalloproteinases (MMPs) [1], [4], [13], [14]. Conversely, the inflammatory mediator nuclear factor κ-B (NF-κB) modulates Fzd mRNA expression and GSK-3β suppression can induce NF-κB-mediated transcription [7], [15]. It has been suggested that modulating the β-catenin pathway in the airway epithelium could have promising impact on airway inflammation and remodeling [4], [9], [14]. However, the involvement of the Wnt/β-catenin signaling pathway in human airway responsiveness remains scarce.

β_2_-adrenoceptor agonists are the most potent known airway smooth muscle relaxants and they have been used for several decades to treat asthma and chronic obstructive pulmonary disease. However, regular use of β_2_-agonists alone may enhance non-specific airway responsiveness and inflammation, thereby deleteriously affecting control of chronic inflammatory airway diseases [16]–[18]. Functional studies have suggested that untoward effect involves cAMP–protein kinases A (PKA) cascade and proinflammatory pathways mediated by NF-κB, leading to airway smooth muscle sensitization, airway neuroinflammation, and disturbance of the epithelial regulation of airway smooth muscle contraction [19]–[23]. Nonetheless, the role of NF-κB and other proinflammatory mediators in this untoward effect remains unclear [24]. Moreover, PKA can activate the canonical Wnt signaling via the Fzd and LRP5/6 phosphorylation [4], [25]. PKA also inhibits the GSK-3β activity, increasing β-catenin independently of the Wnt signaling [26], [27]. In the same way, the Gs_α_ and Gs_βγ_ proteins stimulated by the G-coupled membrane receptors like β_2_-adrenoceptor may modulate the intracytosolic β-catenin [4], [8].

The purpose of this work was to investigate whether the Wnt signaling pathways are involved in the bronchial hyperresponsiveness induced by prolonged exposure (15 hours) to β_2_-adrenoceptor agonists in human airways. Furthermore, we determined the effects of inhibitors or blockers of Wnt signaling on the β_2_-agonist-induced sensitizing effect in human isolated bronchi and we assessed the impact of prolonged exposure to β_2_-agonist on the mRNA expression of genes interacting with Wnt signaling pathways or β_2_-adrenoceptor/cAMP–PKA cascade in complete human bronchi and in primary cultures of human bronchial epithelial cells.

## Methods

### Patients

The study and the consent procedure were approved by the local Ethics Committee (Comité de Protection des Personnes, Ile de France VIII, Boulogne-Billancourt, France). At least one day prior scheduled surgery for lung cancer, patients gave written informed consent that has been recorded in the individual's file. Bronchial tissues were surgically removed from 44 patients (28/16 men/women, 66±11 year of age); all were smokers or ex-smokers. Bronchial segments (inner diameter 2–3 mm) were collected from sites distant from the tumor. The absence of tumor infiltration was retrospectively established in all bronchi.

### Tissue preparations for function and RNA isolation

**Figure 1:**
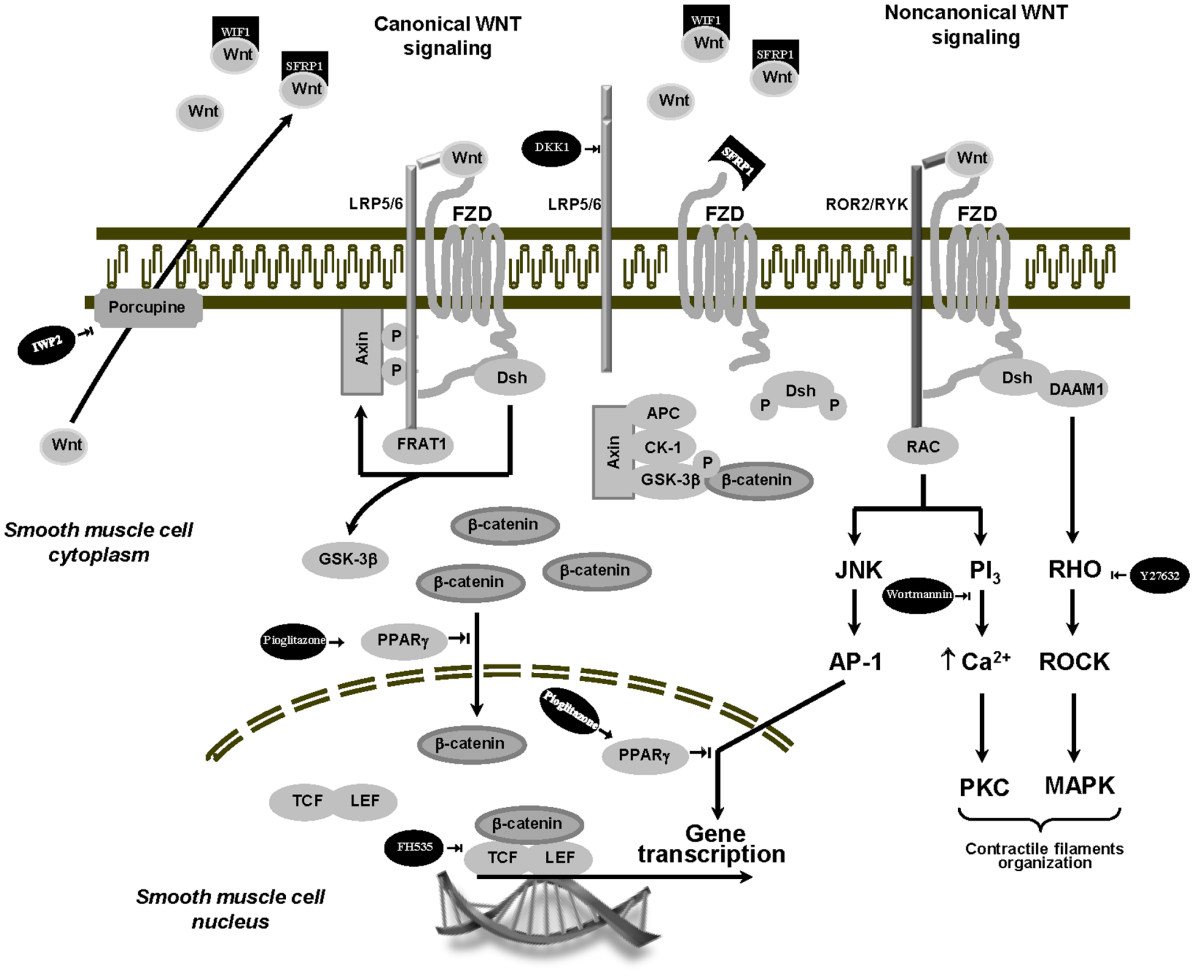
Schematic representation of the canonical (Wnt/β-catenin) and non-canonical (Wnt/Ca^2+^ and Wnt/PCP) signaling pathways. Drugs used in the experiments and their action on Wnt signaling pathways are figured in black. Activation and inhibition are symbolized by 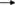 and 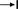, respectively. In the presence of Wnt ligand, Fzd and LRP5/6 form a receptor complex leading to the recruitment of cytosolic proteins Dsh and LRP5/6 phosphorylation. The phosphorylation and partial internalization of LRP5/6 initiate the disruption of the destruction complex (axin, APC, CK-1, and GSK-3β), allowing cytosolic β-catenin accumulation and then translocation into the nucleus, where β-catenin serves as a coactivator of TCF/LEF transcription factors to control gene transcription. Depending on the cellular context, the non-canonical Wnt signaling pathway is stimulated by the binding of Fzd and ROR2/RYK coreceptors. The Wnt/Ca^2+^ pathway induces PKC activation via the intracellular Ca^2+^ increase. The Wnt/PCP pathway activates the small G proteins Rho and the MAPK cascade via the recruitment of the cytosolic proteins Dsh and DAAM1, or, alternatively, triggers activation of JNK leading to the transcription of target genes through AP-1 activation.

Tissues were dissected and washed in oxygenated Krebs-Henseleit solution (composition in mM: 119 NaCl, 4.7 KCl, 2.5 CaCl_2_, 1.2 KH_2_PO_4_, 25 NaHCO_3_ and 11.7 glucose). For function, rings of similar length from the same bronchi were randomly distributed in paired groups. Half of the rings were placed in oxygenated Krebs-Henseleit solution, in parallel the other half was placed in oxygenated Krebs-Henseleit solution with 0.1 µM fenoterol (Boehringer-Ingelheim, Germany) and both were incubated (37°C) for 15 hours as previously described [22]. To investigate the implication of the Wnt-signaling pathways in the fenoterol-induced bronchial hyperresponsiveness, experiments were run in parallel (control and pretreated groups) in the absence or presence of (Fig. 1): 1) two extracellular Wnt pathway modulators, which inhibit Wnt signaling by directly sequestering Wnt ligands and inhibiting both canonical and non-canonical Wnt signaling [3], [4], [8], [13], [25]: secreted fizzled-related protein 1 (SFRP1, 1 µM) or WNT inhibitory factor 1 (WIF1, 1 µM); 2) an extracellular Wnt antagonist, which inhibits Wnt/β-catenin signaling by binding LRP5/6 [1], [2], [4], [28], [29]: Dickkopf protein family 1 (DKK1, 1 µM); 3) an intracellular inhibitor of Wnt processing and secretion, which inactivates a regulator of Wnt secretion (porcupine) [4], [30]: IWP2 (1 µM); 4) a selective Rho-kinase (ROCK1/2) blocker, which inhibits the Wnt-planar cell polarity (PCP) pathway [12]: Y27632 (1 µM); 5) a blocker of phosphoinositide 3-kinases (PI3Ks), which inhibits the Wnt-Ca^2+^ pathway (4, 15): wortmaninn (1 µM); 6) a selective peroxysome proliferator activated receptor-γ (PPAR-γ) agonist, which inhibits both noncanonical Wnt/JNK and canonical Wnt/β-catenin signaling [31], [32]: pioglitazone (1 µM); 7) a modulator of Wnt signaling in the nucleus, which inhibits the recruitment of the co-activators β-catenin (TCF/LEF) [4]: FH535 (0.3 µM). Because of its very short half-life, wortmaninn was added only after 15 hours of incubation at 37°C, immediately to the organ bath after the last change of fresh Krebs−Henseleit solution during the equilibration period. SFRP1, WIF1 and DKK1 were purchased from Abcam (Cambridge, MA). IWP2 came from Tocris Biosciences (Bristol, United Kingdom). Y27632 was purchased from Cayman Chemicals (Ann Arbor, MI). Pioglitazone came from Adipogen (San Diego, CA). Wortmannin and FH535 were purchased from Enzo Life Sciences (Farmingdale, NY). All drugs were dissolved in distilled water except for wortmannin, FH535 and pioglitazone which were dissolved in pure ethanol and DMSO and then diluted in Krebs-Henseleit solution. The final ethanol or DMSO concentration (0.03%) did not alter airway tone or contractility [19]. For RNA isolation, paired bronchial rings were immediately (H0) immersed in RNAlater (Sigma, St. Louis, MO) or were first incubated without or with 0.1 µM fenoterol in oxygenated Krebs-Henseleit solution (37°C) for 15 hours (H15) before immersion in RNAlater. Bronchi were then stored at −80°C until use.

### Tissue preparations for primary culture

Tissues were processed within 24 hours after surgery. Bronchial epithelial cells were harvested using enzymatic isolation procedures to establish primary cultures, as previously described [33]. In brief, bronchi were incubated for 24 hours at 4°C with 0.1% protease in DMEM/Ham's F12 medium. After neutralization of these enzymes by 10% foetal bovine serum, the cells were centrifuged at 800 rpm for 5 min. Cells were plated on home-made permeable collagen supports and cultured at air-liquid interface at 37°C in a humidified atmosphere of 5% CO_2_ in air. The culture medium consisted of DMEM/Ham's F12 medium supplemented with: insulin (5 µg/ml), transferrin (7.5 µg/ml), hydrocortisone (1 µM), endothelial cell growth supplement (2 µg/ml), epithelial growth factor (25 ng/ml), triiodothyronine (30 nM), L-glutamine (1 mM), penicillin/streptomycin (100 µg/ml), gentamycin (50 µg/ml), and amphotericin B (1 µg/ml). The cultures reached confluence between 4 and 10 days after plating and developed a transepithelial potential difference. Then, 200 µl of culture medium with or without 0.1 µM fenoterol was deposited on the apical side of each culture. After 15 hours, epithelial cells were lysed in TRIzol reagent, and then stored at −80°C until use.

### Bronchial function

After 15 hours of incubation with or without fenoterol, the bronchial rings were suspended on hooks in a 5-ml organ baths containing Krebs-Henseleit solution, gassed with 95% O_2_-5% CO_2_ and maintained at 37°C. Each preparation was connected to a force displacement transducer and isometric tension was recorded continuously for analysis (IOX2 software EMKA Technologies, Paris, France). Preparations were suspended with an initial tension of 1.5 g in organ baths [21], [22]. Bronchial rings were maintained in organ bath for 1 hour with washing every 10–15 min to equilibrate and stabilize at a resting tone. Function was assessed by successive additions of endothelin-1 (ET-1), a peptide implicated in chronic inflammatory diseases, to the rings in organ baths (10^−10^ to 10^−7^ M with logarithmic increments) [19]–[22]. ET-1 was purchased from Sigma−Aldrich (St. Louis, MO). After experiments, rings that had been patted dry were weighed.

### RNA isolation and reverse transcriptase – quantitative polymerase chain reaction (RT-qPCR) analysis

The RT-qPCR experiments were performed as described previously [34]. Bronchial rings were crushed and homogenized in TRIzol reagent immediately after dissection, using a TissueLyser LT ball mill (Qiagen Courtaboeuf, France). Total RNA was extracted from bronchus homogenates using TRIzol. The amount of RNA extracted was estimated by spectrophotometry at 260 nm (Biowave DNA; Biochrom, Cambridge, England) and the quality of the preparation was assessed in a microfluidic electrophoresis system (RNA Standard Sensitivity kits for Experion, BioRad, Marnes-la-Coquette, France). After treatment with DNase I (Life Technologies, Saint Aubin, France), 1 µg of total RNA was reverse-transcribed (SuperScript III First-Strand SuperMix kit, Life Technologies). The resulting cDNA was then used for RT-qPCR experiments with TaqMan chemistry (Life Technologies). After initial denaturation at 95°C for 10 min, 20 ng of cDNA were amplified (using Gene Expression Master Mix, Life Technologies) in 40 annealing/extension cycles (95°C for 15 sec and 60°C for 1 min) in a StepOnePlus thermocycler (Life Technologies). The sample's fluorescence was measured after each cycle and the threshold cycle (Ct) of the real-time PCR was defined as the point at which a fluorescence signal corresponding to the amplification of a PCR product was detectable. The reaction volume was 10 µl. The following genes were considered as relevant for the study [1], [6], [7], [10]–[14], [27], [31], [35]: 1) genes related to Fzd receptors: *FZD1-10*; 2) genes related to Wnt proteins: *WNT2, WNT3A, WNT5A, WNT7B, WNT10A*, and *WNT11*; 3) genes related to LRP5/6 co-receptor: *LRP5* and *LRP6*; 4) genes that interact with the cAMP–PKA cascade and the β_2_-adrenoceptor regulation: *SLC9A3R1, PPP2R1A, EP300, CTBP1*, and *FRAT1*; 5) two genes related to PKC, p^38^/JNK or Rho/MAPK signaling pathways: *PKCE* and *GADD45A*; 6) gene related to Wnt signaling pathway regulation: *WISP1*. The expression of transcripts of the relevant genes has been analyzed in the bronchi using a specific TaqMan Array based on predesigned reagents (Assay-on Demand, Life Technologies). In order to validate the extraction of intact cellular mRNA and standardize the quantitative data, three reference genes, hypoxanthine phosphoribosyltransferase (*HPRT1*), glyceraldehydes-3-phosphate dehydrogenase (*GAPDH*) and β-glucuronidase (*GUSB*) were amplified simultaneously.

### Data analysis and statistics

Values are presented as means ± SEM. The results were analyzed using Student's *t* test for paired data and repeated measures ANOVA with Bonferroni adjustments for multiple comparisons (StatView 5.0, SAS Institute, Cary, NC). A value of *P*<.05 was considered statistically significant. Because inadequate statistical power cannot rule out related to small samples, the standardized effect size *d* for the difference between means was calculated to determine whether the observed effect of pretreatment co-incubated with fenoterol was small (|*d*|≥.20), medium (|*d*|≥.50), or large (|*d*|≥.80) according to the Cohen's conventions [36]. Contractile responses were expressed as g/basal tone. The data are expressed in terms of Emax for efficacy and −log EC_50_ (pD_2_) for potency. Emax (g) represented the maximal contraction induced by 0.1 µM ET-1 [19]. ΔEmax represented the difference between Emax obtained with the fenoterol-treated bronchi and Emax obtained with their paired controls. ET-1 potency (−log EC_50_) was derived graphically from the log concentration-effect curves and defined as the negative log of the ET-1 concentration achieving 50% of the maximal effect. Δ(−log EC_50_) represents the difference between −log EC_50_ obtained with the fenoterol-treated bronchi and −log EC50 obtained with their paired controls. The quantitative data obtained from RT-qPCR experiments was expressed as relative expression (2^−ΔCt^) [37] where ΔC_t_ is the difference between the target gene C_t_ and the mean C_t_ of the reference genes.

## Results

### Sample

**Figure 2:**
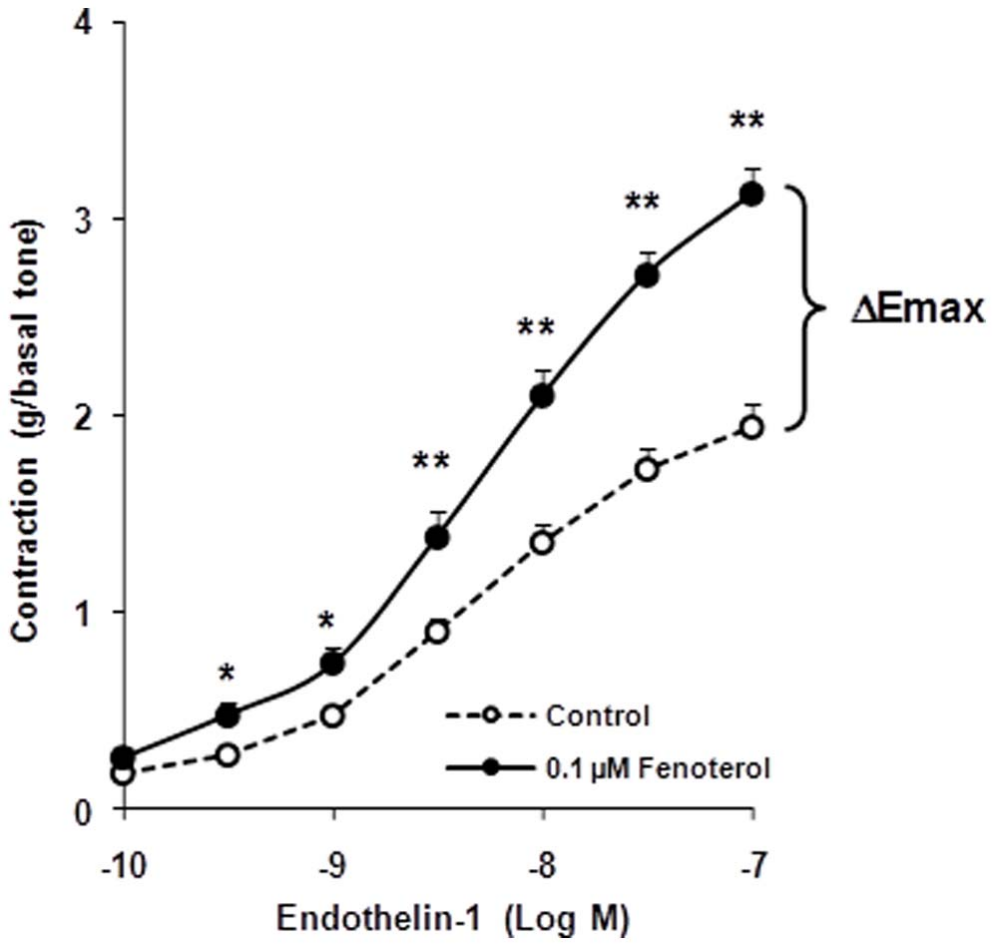
Concentration–response curves for endothelin-1 (ET-1)–induced contraction in human bronchi after 15 hours of incubation with 0.1 µM fenoterol or Krebs–Henseleit solution for the paired control (open circles, control; filled circles, 0.1 µM fenoterol). ΔEmax: difference between maximal induced contractions of fenoterol-pretreated bronchi and paired-control bronchi. Values are means ± SEM (*n* = 44). *P<.01, **P<.001 fenoterol vs paired control.

Mean patted-dry weight of bronchi exposed to fenoterol was comparable with that of their paired controls (28.21±2.18 vs 27.55±1.90 mg, *n* = 44, *P* = 0.76). Fenoterol (0.1 µM, 15 hours) did not change significantly the basal tone of human bronchi before contraction with ET-1 (1.16±0.08 vs 1.08±0.08 g in paired controls, *n* = 44, *P* = 0.39). Incubation of the bronchi with 0.1 µM fenoterol significantly enhanced the ET-1–induced contraction (Figure 2). Emax was 3.12±0.14 g in the presence of fenoterol vs 1.94±0.11 g for the paired–control rings (ΔEmax  =  1.18±0.12 g, *n* = 44, *P*<.0001). −log EC_50_ values was 8.36±0.09 log units in the presence of fenoterol vs 8.27±0.10 log units for the paired–control rings [Δ(−log EC_50_)  =  0.08±0.09, *n* = 44, *P* = 0.37; Fig. 2].

### RT-PCR

**Figure 3:**
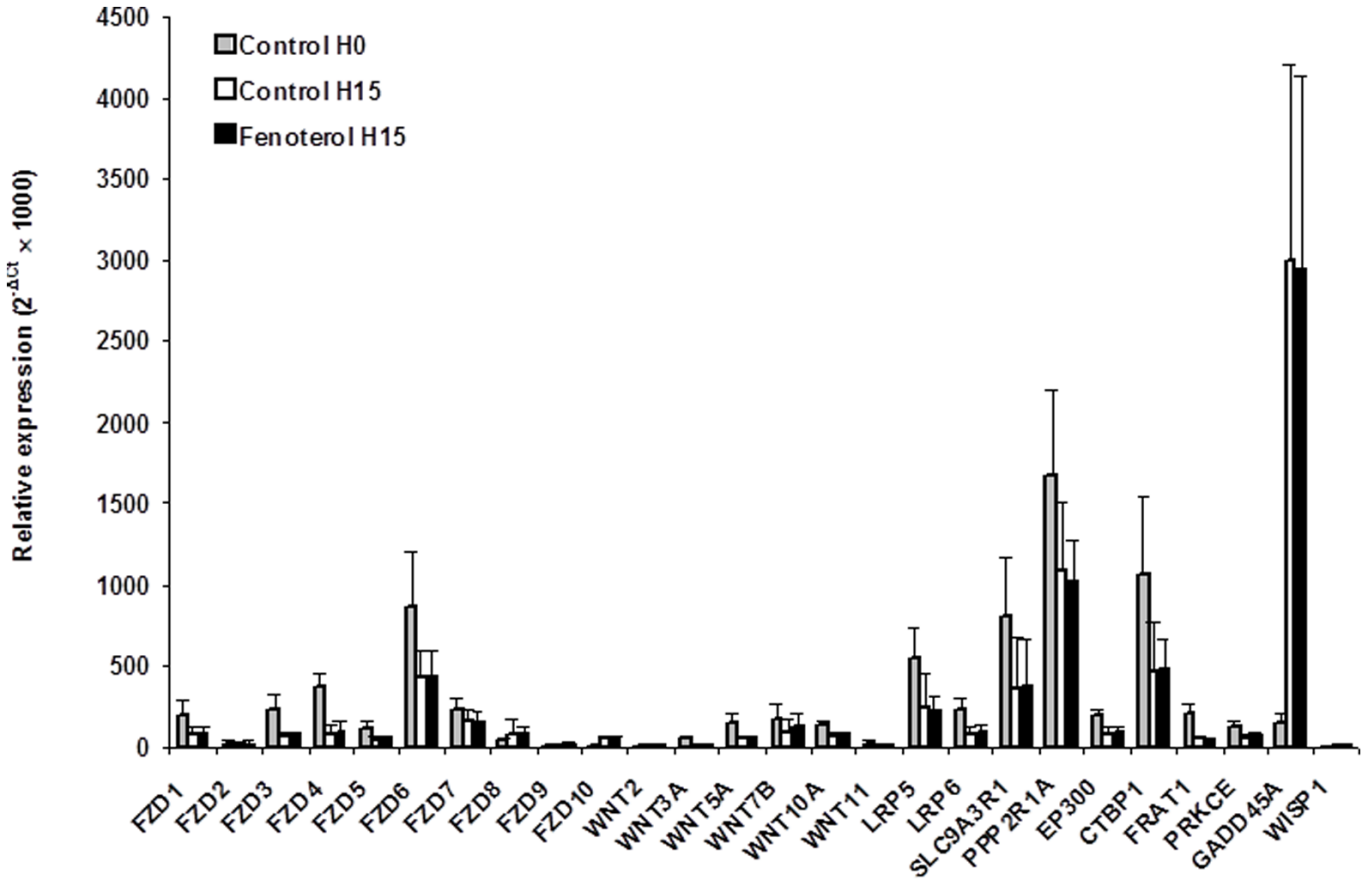
Gene-expression in complete human bronchi at basal state (H0) and after 15 hours of incubation (H15) with 0.1 µM fenoterol or Krebs–Henseleit solution (paired controls). Data are expressed as relative expression (where ΔC_t_ is the difference between the target gene C_t_ and the mean C_t_ of the reference genes). Values are means ± SEM (*n* = 8). No difference between fenoterol and paired controls were statistically significant.

**Figure 4:**
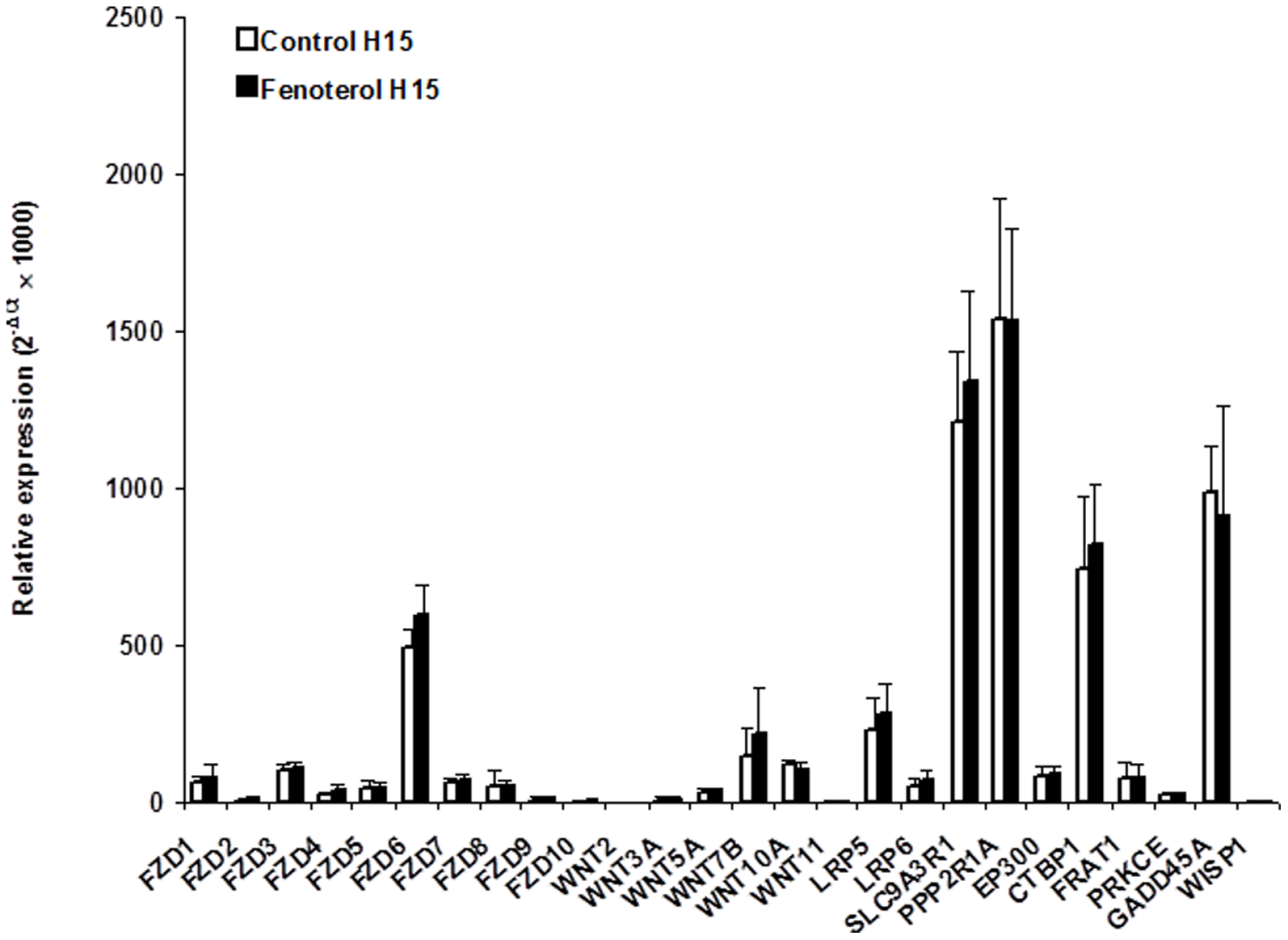
Gene-expression in primary cultures of human airway epithelial cells incubated with or without (paired controls) 0.1 µM fenoterol for 15 hours. Data are expressed as relative expression (where ΔC_t_ is the difference between the target gene C_t_ and the mean C_t_ of the reference genes). Values are means ± SEM (*n* = 6). No difference between fenoterol and paired controls were statistically significant.

The mRNA levels of the 3 housekeeping genes were unaffected by incubation with fenoterol or incubation time (not shown). At basal state (H0), whole human bronchi mainly expressed FZD6 mRNA while the WNTs expression was low, and WIF1 expression remained practically undetectable (Fig. 3). Incubation with Krebs-Henseleit solution for 15 hours led to a rise in GADD45A mRNA expression, a gene involved in p^38^/JNK activation (Fig. 3). Compared to paired tissues incubated with Krebs-Henseleit solution, incubation with 0.1 µM fenoterol for 15 hours did not alter the mRNA expression of genes related to Wnt signaling pathway (FZD1-10, WNT2, WNT3A, WNT5A, WNT7B, WNT10A, WNT11, LRP5/6, and WISP1) or genes interacting with the modulation of cAMP–PKA cascade (SLC9A3R1, PPP2R1A, EP300, CTBP1, and FRAT1) or the p^38^/JNK or Rho/MAPK signaling pathways (PKCE and GADD45A) (Fig. 3). Moreover, FZD6, WNT7B and LRP5 mRNA were mostly expressed by cultured human bronchial epithelial cells (Fig. 4). Compared to paired controls, mRNA expression of the genes of interest did not appear to be changed in fenoterol-treated epithelial cells (Fig. 4).

### Effect of extracellular Wnt inhibitors on fenoterol-sensitization

**Figure 5:**
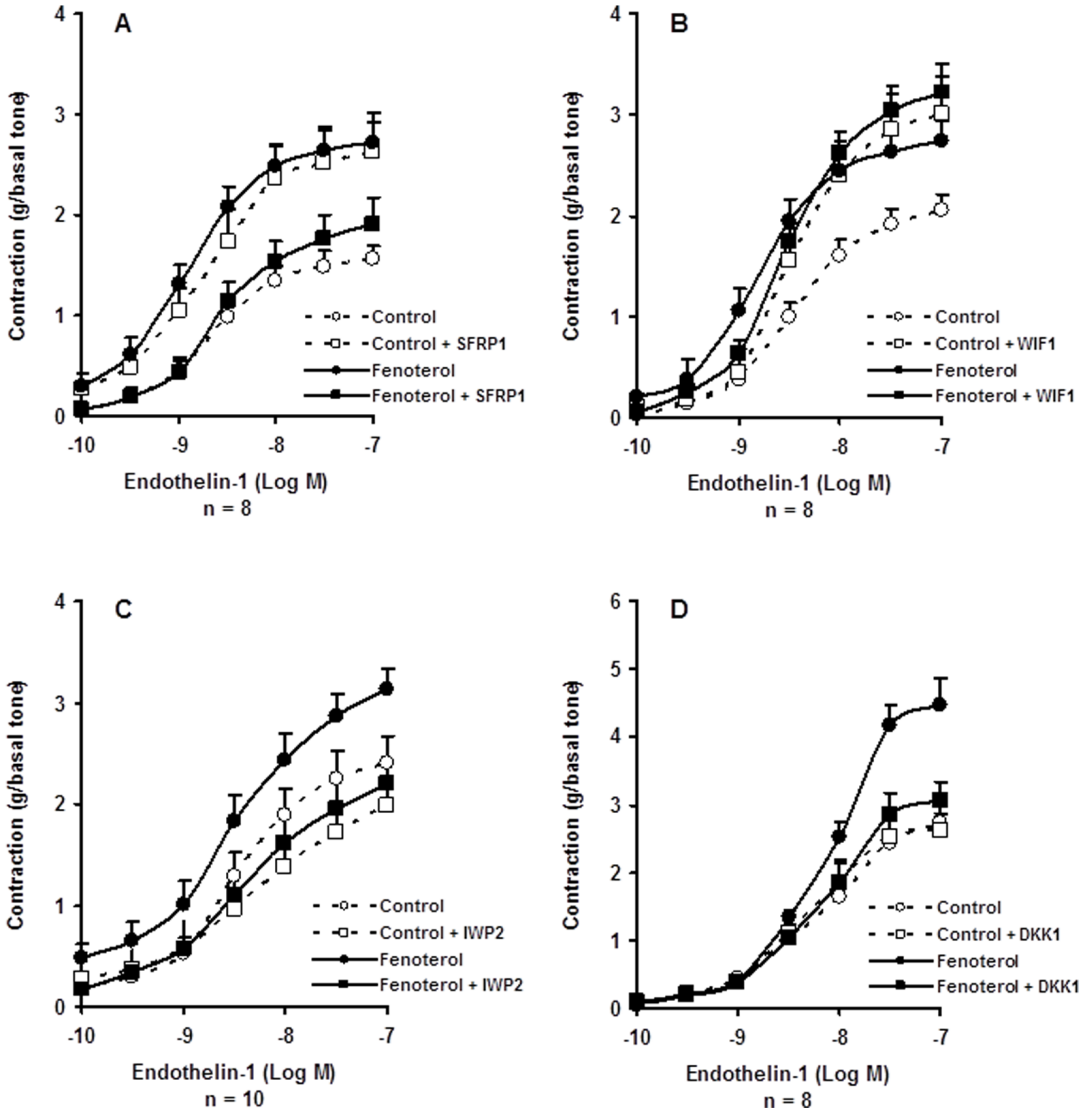
Concentration–response curves for endothelin-1 (ET-1)–induced contraction in human bronchi after 15 hours of incubation with 0.1 µM fenoterol or Krebs–Henseleit solution for the paired control in presence or in absence of: (*A*) SFRP1 (1 µM); (*B*) WIF1 (1 µM); (*C*) IWP2 (1 µM); (*D*) DKK1 (1 µM). Open circles, control; Filled circles, 0.1 µM fenoterol; Open square, control + pretreatment; Filled square, fenoterol + pretreatment. Values are means ± SEM. For comparisons, see Tables 1 and 2.

Blocking the extracellular Wnts or the Fzd receptors by 1 µM SFRP1 or 1 µM WIF1 increased the contractile response to ET-1 of human bronchi (Fig. 5A–B). When the same paired human bronchi were incubated with 0.1 µM fenoterol, co-incubation with SFRP1 abolished the sensitizing effect provoked by fenoterol or SFRP1 exposure (Fig. 5A, Tables 1 and 2) whereas co-incubation with WIF1 did not change significantly the fenoterol-induced sensitizing effect (Fig. 5B, Tables 1 and 2).

**Table 1:** Action of pretreatments on the changes in efficacy (ΔEmax) of endothelin-1 (ET-1) in human isolated bronchi.

Pretreatment 15 hours, 37°C		ΔEmax ET-1, g		Effect size (pretreatment)
	*n*	Fenoterol only	Fenoterol + Pretreatment	*d* [95% CI]
SFRP1 (1 µM)	8	1.16±0.17	−0.72±0.42***	−2.09 [−3.17 – −0.78]
WIF1 (1 µM)	8	0.68±0.22	0.22±0.40	−0.51 [−3.17 – 0.51]
IWP2 (1 µM)	10	0.73±0.10	0.24±0.21	−0.95 [−1.83 – −0.01]
DKK1 (1 µM)	8	1.81±0.26	0.43±0.30**	−1.75 [−2.79 – −0.52]
Wortmaninn^†^ (1 µM)	8	0.67±0.16	−0.22±0.25*	−1.51 [−2.53 – −0.33]
Y27632 (1 µM)	10	1.24±0.30	−0.01±0.15***	−1.68 [−2.62 – −0.62]
Pioglitazone (1 µM)	11	0.90±0.17	−0.13±0.19***	−1.74 [−2.65 – −0.71]
FH535 (0.3 µM)	9	0.76±0.21	0.02±0.19**	−1.23 [−2.17 – −0.17]

**Figure 6:**
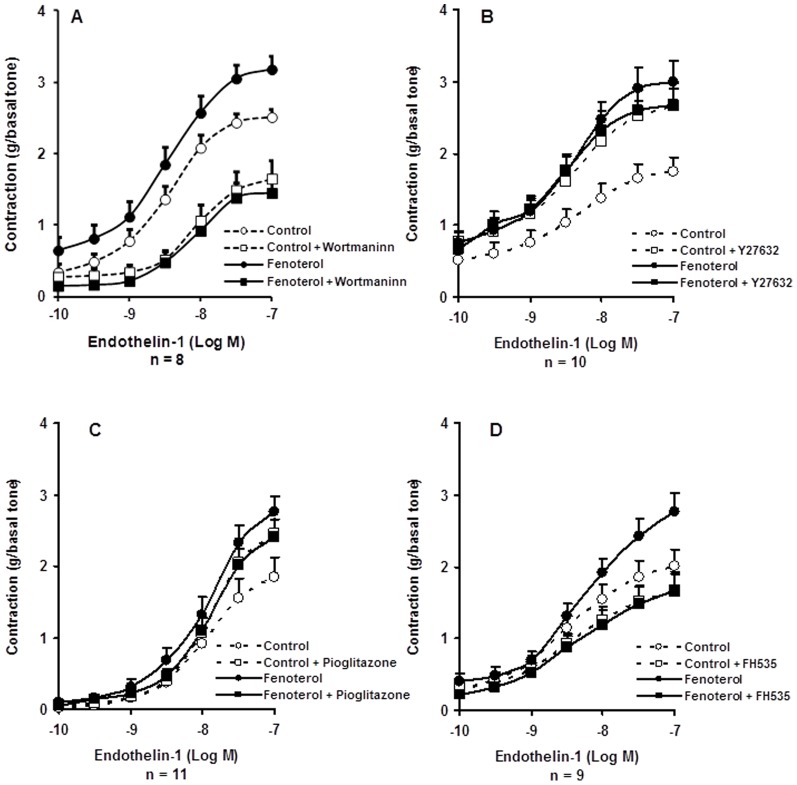
Concentration–response curves for endothelin-1 (ET-1)–induced contraction in human bronchi after 15 hours of incubation with 0.1 µM fenoterol or Krebs–Henseleit solution for the paired control in presence or in absence of: (*A*) Wortmaninn (1 µM); (*B*) Y27632 (1 µM); (*C*) Pioglitazone (1 µM); (*D*) FH535 (0.3 µM). Open circles, control; Filled circles, 0.1 µM fenoterol; Open square, control + pretreatment; Filled square, fenoterol + pretreatment. Values are means ± SEM. For comparisons, see Tables 1 and 2.

Values were determined in paired human bronchi after incubation with 0.1 µM fenoterol or oxygenated Krebs–Henseleit solution (control) for 15 hours at 37°C with or without pretreatment co-incubated with fenoterol (Fig. 5–6). Emax (g) represented the maximal contraction induced by 0.1 µM ET-1. ΔEmax represented the difference between Emax obtained with the fenoterol-treated bronchi and Emax obtained with their paired controls. The observed effect of pretreatment co-incubated with fenoterol is small (|*d*|≥.20), medium (|*d*|≥.50), or large (|*d*|≥.80) according to the Cohen's conventions [36]. The 95% CI for *d* consists of the uncertainty around the real effect of pretreatment. ^†^Except for wortmaninn which was added in organ bath before concentration-response curve for ET-1. *P<.05, **P<.01, ***P<.001 with vs without pretreatment.

**Table 2:** Action of pretreatments changes in potency (Δ–log EC_50_) of endothelin-1 (ET-1) in human isolated bronchi.

Pretreatment 15 hours, 37°C		Δ(–log EC_50_) ET-1, log unit		Effect size (pretreatment)
	*n*	Fenoterol only	Fenoterol + Pretreatment	*d* [95% CI]
SFRP1 (1 µM)	8	0.46±0.17	−0.20±0.14***	−1.53 [−2.55 – −0.34]
WIF1 (1 µM)	8	0.45±0.17	0.12±0.19	−0.62 [−1.59 – 0.41]
IWP2 (1 µM)	10	0.15±0.12	0.20±0.21	0.09 [−0.79 – 0.97]
DKK1 (1 µM)	8	−0.08±0.14	−0.18±0.14	−0.26 [−1.23 – 0.74]
Wortmaninn^†^ (1 µM)	8	0.08±0.09	0.33±0.16	0.67 [−0.37 – 1.63]
Y27632 (1 µM)	10	0.20±0.32	−0.08±0.23	−0.32 [−1.19 – 0.58]
Pioglitazone (1 µM)	11	0.33±0.18	0.03±0.09	−0.63 [−1.46 – 0.25]
FH535 (0.3 µM)	9	−0.36±0.21	−0.24±0.14	−0.22 [−0.71 – 1.14]

Values were determined in paired human bronchi after incubation with 0.1 µM fenoterol or oxygenated Krebs–Henseleit solution (control) for 15 hours at 37°C with or without pretreatment co-incubated with fenoterol (Fig 5–6). ET-1 potency (−log EC_50_) was derived graphically from the log concentration-effect curves and defined as the negative log of the ET-1 concentration achieving 50% of the maximal effect. Δ(−log EC_50_) represents the difference between −log EC_50_ obtained with the fenoterol-treated bronchi and −log EC50 obtained with their paired controls. The observed effect of pretreatment co-incubated with fenoterol is small (|*d*|≥.20), medium (|*d*|≥.50), or large (|*d*|≥.80) according to the Cohen's conventions [36]. The 95% CI for *d* consists of the uncertainty around the real effect of pretreatment. ^†^Except for wortmaninn which was added in organ bath before concentration-response curve for ET-1. *P<.05, **P<.01, ***P<.001 with vs without pretreatment.

### Effect of the inactivation of Wnt Secretion on fenoterol-sensitization

Inactivating the Wnt secretion by co-incubation with 1 µM IWP2 had no significant effect on the rise in maximal airway contraction to ET-1 elicited by fenoterol exposure for 15 hours in 10 human bronchi (Fig. 5C, Tables 1 and 2).

### Effect of a LR5/6 inhibitor on fenoterol-sensitization

Compared to paired controls, co-incubation with 1 µM DKK1 significantly decreased the fenoterol-induced rise in efficacy to ET-1 in human bronchi (Fig. 5D, Tables 1 and 2).

### Effect of intracellular Wnt/Ca^2+^ and Wnt/PCP pathways inhibitors on fenoterol-sensitization

In absence of fenoterol, 1 µM wortmaninn and 1 µM Y27632 produced opposite effects on the bronchial contraction to ET-1 (Fig. 6A–B). Blockage of PI3Ks by 1 µM wortmaninn or blockage of Rho-kinases by 1 µM Y27632 abolished the rise in airway maximal response to ET-1 provoked by fenoterol exposure for 15 hours (Fig. 6A–B, Tables 1 and 2).

### Effect of nuclear modulators of Wnt signaling on fenoterol-sensitization

As seen in Fig. 6C and Tables 1 and 2, co-incubation with a non-selective inhibitor of canonical and non-canonical Wnt signaling pathways, pioglitazone (1 µM), decreased the fenoterol-induced rise in airway maximal contraction to ET-1 in human bronchi. Similarly, inhibiting the canonical Wnt/β-catenin pathway by co-incubation with 0.3 µM FH535, abolished the sensitization induced by fenoterol exposure for 15 hours (Fig. 6D, Tables 1 and 2). Pioglitazone and FH535 had opposite effects on the bronchial contraction to ET-1 (Fig. 6C–D).

## Discussion

This study shows that fenoterol, a β_2_-agonist with high intrinsic efficacy, sensitized human bronchi at least in part via the Wnt/β-catenin signaling, notably the LRP5/6 co-receptor and the nuclear recruitment of TCF/LEF. Conversely, fenoterol exposure did not change the Wnt secretion and the mRNA expression of genes interacting with the regulation of Wnt proteins, Fzd-receptors, LRP5/6 co-receptor, PKC, p^38^/JNK or Rho/MAPK, and cAMP–PKA cascade. Moreover, the present study confirms the implication of p^38^/JNK and Rho/MAPK signaling pathways in the fenoterol-induced sensitizing effect [19] which could also be explained by the involvement of the non-canonical Wnt signaling. Taken together, our observations suggest that 15 hours of fenoterol exposure provokes the recruitment of Wnt/β-catenin signaling via the LRP5/6 co-receptor and/or Fzd-receptors phosphorylation, regardless of the Wnt secretion. In this way, the involvement of Wnt/β-catenin signaling in the fenoterol-sensitization suggests a mechanism of functional selectivity or biased agonism in connection with the β_2_-adrenoceptor stimulation [38]–[40]. However, the stimulation of Wnt/β-catenin signaling pathway by fenoterol does not fully account for the fenoterol-induced sensitizing effect. Indeed, previous reports have shown that untoward effect was mediated by various pro-inflammatory mediators activated via the prolonged stimulation of the β_2_-receptor [19]–[23]. Pertinently, co-incubation with anti-inflammatory drugs or proinflammatory mediator receptor antagonist such as corticosteroids or non-steroidal anti-inflammatory drugs did not fully inhibit the fenoterol-sensitization [19], [20]. The involvement of the Wnt/β-catenin signaling pathway, which is not directly influenced by these drugs, brings a better understanding of previous published data.

In the classical pharmacological model, agonist binding to seven-transmembrane receptor such as β_2_-adrenoceptor provokes changes in receptor conformation that results in the dissociation of heterotrimeric G proteins into Gs_α_ and Gs_βγ_ subunits leading to the stimulation of cAMP−PKA cascade and the G protein-coupled receptor kinases which mediate receptor desensitization. However, in the last decade, this classical model became incomplete [38]–[40]. The functionnal selectivity or biased agonism is a property of a ligand-receptor complex where seven-transmembrane receptors can adopt multiple active conformations that can result in different signaling pathways activation. Depending on the cellular context and the intrinsic activity of a specific ligand, β_2_-adrenoceptor stimulation can switch coupling from Gs to Gi heterotrimeric proteins or activate intracellular signaling pathways (MAPK, PI3K, PKC) via β-arrestin, a G protein-coupled receptor kinase [38]. The Gi_α/0_ subunit can also interact with the LRP5/6-Fzd receptor complex thus activating the canonical Wnt pathway [41] Moreover, both G_α_ and G_βγ_ subunits have been shown to promote canonical Wnt signaling via Dsh, a protein involved in the Wnt/β-catenin pathway by interacting with the initiation of LRP5/6 phosphorylation by the axin-GSK-3β complex (Fig. 1) [4], [8]. It has also been shown that activated-phosphorylated forms of LRP5/6 may trigger Wnt/β-catenin signaling in an Fzd- and Dsh-independent manner [8]. In line with these findings, we showed herein that DDK1, a blocker of LRP5/6, inhibited the fenoterol-induced sensitization of human bronchi. Intriguingly, we also found that SPRP1 and WIF1, two extracellular Wnt scavengers, increased the contraction of human bronchi in absence of fenoterol whereas only SFRP1 inhibited the fenoterol-induced sensitizing effect. The sensitizing effect provoked by WIF1 in absence of fenoterol can be explained by the fact that the ROR2/RYK co-receptor contains a WIF-binding sequence, which may facilitate the non-canonical Wnt pathway activation [4]. Unlike WIF1, SFRP1 can also act by binding to Fzd receptors [42], thereby indicating prolonged β_2_-adrenoceptor stimulation may lead to mimic Wnt/β-catenin signaling activation by stimulating indirectly LRP5/6 or Fzd receptors [4], [8]. We hypothesize that cAMP−PKA cascade induces phosphorylation and activation of LRP5/6 and Fzd receptors in a Wnt ligand-independent manner as suggested by the lack of significant effect of IWP2 and WIF1 on the feneterol-induced sensitization. The absence of changes in mRNA expression of genes modulating Wnt proteins and Fzd receptors after fenoterol exposure supports this hypothesis. In our in vitro model of fenoterol-induced Wnt/β-catenin activation, the direct participation of the GSK-3β inhibition provoked by the cAMP−PKA cascade [43] is unlikely given that wortmaninn, a PI3K inhibitor which activates GSK-3β [15], abolished the airway hyperesponsiveness elicited by fenoterol exposure. Similarly, pioglitazone, a PPAR-γ agonist also known as a GSK-3β activator [44], inhibited the fenoterol-sensitizing effect. By contrast, we cannot completely exclude the activation of the non-canonical Wnt signaling by β-arrestin [45] since wortmaninn and Y27632, two blockers of the Wnt/PCP and Wnt/Ca^2+^ pathways, abolished the fenoterol-sensitizing effect. However, we have previously shown that the MAPK and PKC pathways can be triggered by other intracellular mediators that non-canonical Wnt signaling [19] and the involvement of the non-canonical Wnt signaling cannot explain the effects of specific canonical Wnt inhibitors as DDK1 or FH535 in the present study.

In this study, we found that pioglitazone and FH535, two nuclear blockers of Wnt signaling, abolished the fenoterol-sensitizing effect. Pioglitazone acts by stimulating PPAR-γ, a nuclear hormone receptor which inhibits both canonical and non-canonical Wnt signaling pathways [2], [31], [44], [46]. PPAR-γ is expressed in airway smooth muscle and epithelial cells and is induced by cAMP [22]. However, the mechanism of action of pioglitazone may also involve the simulation of the voltage-gated Ca^2+^ channels on the airway smooth muscles [32], which would explain why pioglitazone increased the bronchial contraction in absence of fenoterol. FH535 is a specific inhibitor of Wnt/β-catenin signaling, indicating fenoterol-sensitization mainly involves the canonical Wnt pathway. Moreover, FH535 has the effect of blocking the transcription of β-catenin-dependent genes, suggesting that the fenoterol-sensitizing effect was mediated, at least in part, by gene transcription. Although we couldn't find herein an increase in mRNA expression of genes regulating Wnt signaling pathways, we have previously shown that fenoterol up-regulated the mRNAs of cytokines/proteins implicated in the recruitment of T and B cells or the activation and proliferation of bronchial epithelial cells (CCL20/MIP-3α, FOXA2, PPAR-γ) in human isolated bronchi and in cultured epithelial cells [32]. Moreover, the Wnt/β-catenin signaling directly or indirectly increases the mRNA expression of various genes such as NF-κB, CXCL8/IL-8, COX2, and MMP [4], [10], [15]; this is consistent with previous findings showing that the sensitizing effect of fenoterol was inhibited by gliotoxine (a NF-κB inhibitor) or indomethacin (a COX-2 inhibitor) [19].

The major limitation of the present study is its almost exclusively functional design with the use of classical pharmacological means (inhibitors/blockers). Given that measurement of mRNA expression could not exactly reflect the changes of Wnt excretion, our results should be confirmed at the protein expression level, and extrapolating our data to clinical outcomes was beyond the scope of our study. However, it is uncertain that investigating the Wnt excretion during fenoterol incubation for 15 hours at 37°C could validate our functional results because of the action of the epithelial peptidases, limiting the half life of excreted proteins [47]. In addition, the very limited expression of Wnt mRNA observed herein and the absence of significant effect of IWP2 or WIF1 on fenoterol-sensitization may be taken as indirect evidence of the stimulation of Wnt/β-catenin signaling pathway at the intra-cytosolic level by cAMP−PKA cascade.

## Conclusion

Collectively, our pharmacological investigations indicated that fenoterol may activate, at least in part, the Wnt/β-catenin signaling pathway in human isolated bronchi, suggesting a mechanism of biased agonism. To a clinical point of view, these findings highlight the interest of Wnt signaling pathways in inflammatory airway diseases. Future experiments based on the results of the present study will be needed to determine the impact of prolonged fenoterol exposure on the extra- and intracellular Wnt signaling pathways at the protein expression level.
